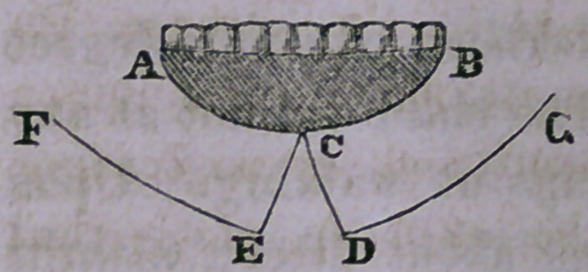# Cook County Hospital Surgical Clinic

**Published:** 1866-04

**Authors:** Geo. K. Amerman

**Affiliations:** Attending Surgeon


### HOSPITAL REPORTS.

*Cook County Hospital Surgical Clinic.*—By Dr. Geo. K. Amerman, Attending Surgeon.

(Reported for the Chicago Medical Journal.)

Gentlemen,—To-day we meet you for the first time under the new administration of this Hospital, to inaugurate, under many temporary inconveniences incident to a new enterprise of this character, our clinical course of study. The reorganization of this Hospital, under its present management, makes it a permanent institution, and we now have every means afforded for clinical instruction, and hope to be able to present to you, from this time forth, the cases at the bedside, in such a way as to make you familiar with disease and accident of every kind.

We are glad to see so many of you on this occasion, and we welcome you to the wards of this institution. To-day we commence our regular course of clinics, to be continued throughout the entire year; and as the executive officers of the Hospital are willing to afford every facility to the Medical Board for clinical instruction, we trust we shall succeed in our endeavor to make the course interesting and instructive to each one of you.

With these brief remarks, let us turn our attention to the patient now before us.

He states that he is 53 years old, and has always enjoyed good health. That he is by occupation a sailor, and that two years ago he fell from the rigging of a ship and struck on the left side, receiving a severe contusion over the anterior tibial region of that side. The injury, however, was not so serious as to prevent him from pursuing his ordinary avocation, and supposing it only a temporary inconvenience, he paid very little attention to it, using only simple domestic remedies as suggested by his friends.

About six months after the occurrence of this injury, a swelling made its appearance over the anterior middle third of the left tibia, and continued to increase for several weeks, and finally, opened and discharged a large quantity of unhealthy pus.

From that time to the present, a year and a half, the limb has been a constant source of trouble; and for the last six months a large ulcer has existed over the original seat of the injury, occupying the lower two-thirds and nearly surrounding the entire limb.

At present the leg is flexed upon the thigh, and cannot be extended beyond a right angle. It is swollen, painful at times, and entirely useless. The ulcer has an unhealthy, livid appearance. The surrounding soft parts are œdematous. His general health is good.

The length of time this disease has existed—its traumatic origin and gradual progress—render it very probable that the original difficulty commenced in the bone and periosteum, and that we now have osteitis, continuing for two years, and finally giving rise to inflammation and ulceration of the soft parts.

With this view of the case, what can we do toward curing the patient? What medicines control or exercise any influence over this disease? What local means will aid in healing this large ulcer, and restoring the proper functions of the limb?

So far as I know, there are no means, constitutional or local, which promise any permanent benefit in a case of this kind. Osteitis, at this stage, is entirely beyond the reach of remedial means. The gradual hypertrophy of the osseous tissue, which always ensues from this long continued over-action of the part, and the interstitial molecular changes which result therefrom, cannot be influenced, in any degree, by the administration of medicine. After the bone has become enlarged, the periosteum thickened, the soft parts extensively involved, and the limb contracted and useless, the only course to be pursued is the removal of the diseased part; and in this case, amputation of the leg is the only thing that will afford any reasonable expectation of a cure.

The patient is anxious to have the part removed, and after consulting with the surgical staff of the Hospital, we have decided upon the operation, and will now proceed to remove the limb about four inches below the knee-joint.

The first thing after deciding to perform an operation, is to select and arrange the instruments necessary for its proper performance. These should be placed on a small bench or stool, and arranged in just the order they will be required, so that without any trouble or inconvenience, you can put your hand on them at any time.

The next thing, is, to arrange the bed or table for the patient to lie on during the operation. This should be placed in a light, airy apartment, should be about two and a half feet high, and stand firm. It should be covered with two or three folds of clothing, so as not to be too hard, and, if convenient, this covered with oil cloth, to prevent soiling.

These arrangements all complete, let the patient remove his outside clothing, and after fixing his position fairly on the bed, administer the anæsthetic. As soon as the patient is fully under the influence of the ether, you carefully apply the tourniquet, which is always safer and better than trusting to the fingers of an assistant, draw the patient down to the edge of the bed, and directing your assistant to hold the limb, you proceed, slowly and carefully, to its removal.

(In this case we shall adopt the old antero-posterior flap method, and as that operation is familiar to you all, we will not describe the steps of the operation.)

After the limb is removed, proceed to apply the necessary dressing. These should always be very simple and light. First, carefully secure every bleeding vessel, then stitch the flaps together, accurately adjusting their edges with as many interrupted sutures as are necessary for that purpose, and placing over this three or four thicknesses of muslin, wet in cold water, as a compress, and over the whole a roller bandage completes the dressing.

The patient will now be removed to the ward, a full anodyne administered, and at our next clinic we will see the progress and result of the case.

January 27. We will first examine the patient whose leg was amputated a week ago. You perceive he appears quite comfortable. The pulse is only 80, appetite good, bowels regular, and he rests well at night. Since the operation there has been no constitutional disturbance, and the only treatment consisted of an occasional anodyne at night. The wound united by adhesion throughout one-half of its extent, and the remainder is closing by granulation. It looks well, and is, for the most part, free from pain. The only dressing is a light compress and roller to steady the parts.

Here we have the specimen after its removal, and it shows, in a remarkable manner, the changes produced in the osseous tissue by long-continued inflammation. The soft parts and periosteum have been removed, and you perceive, in the first place, the increase of size, the tibia being nearly a third larger than normal. With this hypertrophy there is also an increase of weight. This tibia weighs at least twice as much as a healthy bone should weigh. And again, the texture is changed; instead of a layer of compact tissue surrounding the cancellous texture and medullary canal, the whole thickness of this bone is composed of a dense hard tissue almost like ivory: *eburnated*, as it is called. And lastly, the surface is everywhere rough and uneven, with numerous processes and tuberosities projecting from it and extending across the interosseous space, which become firmly united to the fibula. These are sometimes called osteophytes.

These changes of size, weight, texture and form are all the result of long continued inflammatory action of the part; and from their character and the nature of the tissue involved, we see how inefficient all medication would prove, and how necessary it becomes to remove the whole of the diseased part.

(This patient was discharged, cured, February 26th, with a good stump.)

The next patient to whom we will direct our attention, has an affection of an entirely different character, and one of very great interest and importance.

He says he is 65 years old, and has always enjoyed good health. That about two years ago a small hard swelling made its appearance on the middle of the lower lip, which at first attracted very little attention, and seemed of trivial importance.

Soon after its appearance, however, it ulcerated and assumed a much more serious form, the ulceration spreading rapidly and involving the surrounding parts. This unexpected development and progress of the disease induced him to consult several surgeons in reference to it, who advised various applications which were assiduously used, but without any apparent benefit. A week ago he was admitted into the Hospital and is very anxious to have something done for relief.

His general health is excellent, and the only difficulty is located in the lower lip, which, as you see, is the seat of this large, ragged, unhealthy-looking ulcer. The whole lip is involved in this disease, and a large portion of it has been entirely destroyed.

The base of this ulcer is indurated and covered with large, flabby, greyish granulations, surrounded by a jagged, irregular edge. It discharges a fœtid ichorous matter mixed with saliva. The surrounding soft parts and neighboring lymphatics are healthy.

The first question that arises in this case relates to the character of this ulceration, which the patient states has existed for nearly two years. Very evidently this is not a common simple sore, and will not yield to ordinary treatment. He has already made diligent use of various local applications, without receiving a particle of benefit, and if we expect to succeed any better, we must first thoroughly understand the nature of the disease.

So far as we can judge from the history of the case, this affection originated without any assignable cause, and this fact, connected with its first appearing as a small tubercle situated on the lower lip, are suspicious circumstances of a malignant nature. Soon after its appearance this swelling ulcerated, and from that day to the present it has rapidly increased in size. These additional facts, the rapid progress of the disease, its tendency to involve surrounding parts, its persistency under treatment, together with those already mentioned, its originating spontaneously as a small tubercle on the lower lip, and withal its present appearance, leave no doubt as to its being the form of malignant disease commonly designated Epithelial Cancer.

Taking this view of the case, then, the next question is, what can we do in the way of treatment? Local applications have already been thoroughly tested and found useless, and constitutional means promise no better success. The experience of the past shows that when the general health is good, the different functions of the body active, with no cachexial symptoms present, internal medication exercises no influence over the progress of any form of cancer, and in every case the only question in reference to the treatment, is regarding the propriety of operating for its removal. In this case an operation is the only resource that promises any benefit whatever, and we must either abandon the patient to his fate, or excise every particle of this diseased mass. The great objection against operating in cancerous affections, is their liability to return and pursue even a more rapid course than if left to themselves, and hence the question of excision becomes a very serious one, and should in every instance be carefully considered, and all the surrounding circumstances taken into account before deciding what course to adopt. In this case the patient is anxious to have the part removed, and after consulting with the surgical staff of the Hospital, it has been decided to excise the entire lower lip, and construct from the surrounding healthy parts as good a substitute as possible. We will now administer the anæsthetic and proceed to perform the operation.

The methods adopted by different surgeons for the formation of a new lip are various, and many of them quite complicated and difficult to perform; but the one that we shall adopt is the common operation first used by Dr. Mutter, I believe, which is easy of execution, makes a good looking lip, and is generally successful.

The annexed cut shows the different incisions and steps of the operation.

The first incision, A B, extends from one angle of the mouth to the other, circumscribing the whole of the diseased part, and should go through the entire thickness of the lip in the healthy surrounding tissue, including every particle of the diseased mass. You next proceed to form a new lip from the surrounding healthy parts by making two other incisions, the first, C D, extending from the centre of the chin downwards and a little outward, to near the base of the lower jaw; and the second, D G, extending from the lower extremity outwards and a little upward, to near the angle of the jaw. The same incisions are made on both sides. These incisions form two quadrilateral flaps, and remaining between these you perceive a triangular portion of healthy tissue. After detaching the flaps from the jaw, you elevate them so that their lower angles, D G, come nearly to a level with the point of this triangular healthy tissue at C, and secure them in that position by a twisted suture, passing through their lower border and through this healthy triangular part. Other sutures are now applied at various points, adapting the parts as nicely as possible with a few strips of adhesive plaster, a compress and light roller.

The patient will now be removed to the ward, an anodyne administered, and at our next clinic we will see the result.

Feb. 13. Patient discharged, cured, with a good lip and very little deformity.

## Figures and Tables

**Figure f1:**
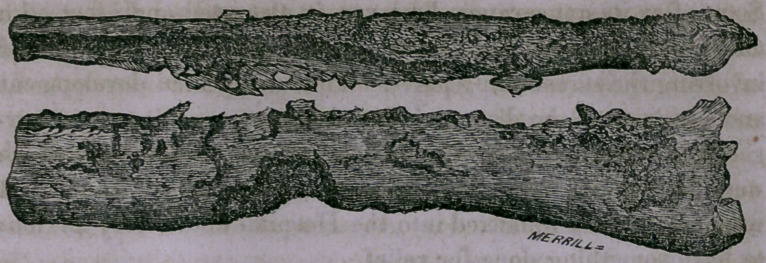


**Figure f2:**